# Central autonomic network dysfunction and plasma Alzheimer’s disease biomarkers in older adults

**DOI:** 10.1186/s13195-024-01486-9

**Published:** 2024-06-08

**Authors:** Trevor Lohman, Arunima Kapoor, Allison C. Engstrom, Fatemah Shenasa, John Paul M. Alitin, Aimee Gaubert, Kathleen E. Rodgers, David Bradford, Mara Mather, S. Duke Han, Elizabeth Head, Lorena Sordo, Julian F. Thayer, Daniel A. Nation

**Affiliations:** 1 University of Southern California, Leonard Davis School of Gerontology, Los Angeles, CA USA; 2 Department of Psychological Science, University of California, Irvine, Irvine, CA USA; 3 Center for Innovations in Brain Science, Department of Pharmacology, University of Arizona, Tucson, AZ USA; 4 Department of Psychology, University of Southern California, Los Angeles, CA USA; 5 Department of Pathology and Laboratory Medicine, University of California, Irvine, Irvine, CA USA; 6 Keck School of Medicine, University of Southern California, Los Angeles, CA USA

**Keywords:** Central autonomic network, Alzheimer’s Disease, Neurofilament light chain, Glial fibrillary acidic protein, Aβ_42/40_

## Abstract

**Background:**

Higher order regulation of autonomic function is maintained by the coordinated activity of specific cortical and subcortical brain regions, collectively referred to as the central autonomic network (CAN). Autonomic changes are frequently observed in Alzheimer’s disease (AD) and dementia, but no studies to date have investigated whether plasma AD biomarkers are associated with CAN functional connectivity changes in at risk older adults.

**Methods:**

Independently living older adults (*N* = 122) without major neurological or psychiatric disorder were recruited from the community. Participants underwent resting-state brain fMRI and a CAN network derived from a voxel-based meta-analysis was applied for overall, sympathetic, and parasympathetic CAN connectivity using the CONN Functional Toolbox. Sensorimotor network connectivity was studied as a negative control. Plasma levels of amyloid (Aβ_42_, Aβ_40_), neurofilament light chain (NfL), and glial fibrillary acidic protein (GFAP) were assessed using digital immunoassay. The relationship between plasma AD biomarkers and within-network functional connectivity was studied using multiple linear regression adjusted for demographic covariates and Apolipoprotein E (*APOE*) genotype. Interactive effects with *APOE4* carrier status were also assessed.

**Results:**

All autonomic networks were positively associated with Aβ_42/40_ ratio and remained so after adjustment for age, sex, and *APOE4* carrier status. Overall and parasympathetic networks were negatively associated with GFAP. The relationship between the parasympathetic CAN and GFAP was moderated by *APOE*4 carrier status, wherein *APOE4* carriers with low parasympathetic CAN connectivity displayed the highest plasma GFAP concentrations (*B* = 910.00, *P* = .004). Sensorimotor connectivity was not associated with any plasma AD biomarkers, as expected.

**Conclusion:**

The present study findings suggest that CAN function is associated with plasma AD biomarker levels. Specifically, lower CAN functional connectivity is associated with decreased plasma Aβ_42/40_, indicative of cerebral amyloidosis, and increased plasma GFAP in *APOE4* carriers at risk for AD. These findings could suggest higher order autonomic and parasympathetic dysfunction in very early-stage AD, which may have clinical implications.

**Supplementary Information:**

The online version contains supplementary material available at 10.1186/s13195-024-01486-9.

## Background

The higher order regulation of autonomic function is controlled by a network of interconnected cortical and subcortical brain regions that maintain homeostasis via respiratory, cardiovascular, digestive, and endocrine system modulation [1, 2]. This regulatory network is referred to collectively as the central autonomic network (CAN). Early descriptions of the CAN in humans included the insular cortex, amygdala, hypothalamus, periaqueductal gray matter, parabrachial complex, nucleus of the tractus solitarius, and ventrolateral medulla [1]. Since these initial descriptions, functional neuroimaging studies have continued to shed light on the complex, context-dependent nature of central autonomic processing, with studies consistently identifying the left amygdala, right anterior insula, left posterior insula, and midcingulate cortices as forming the core of the human CAN [3].

Alzheimer’s disease (AD) affects cortical and limbic regions involved in the CAN [4], and AD patients commonly exhibit peripheral autonomic dysfunction that tracks with disease severity, beginning during early-stage cognitive impairment [5] and progressively worsening in mild- to moderate-stage AD dementia [6, 7]. It has been hypothesized that peripheral autonomic changes observed in AD are likely a consequence of neurodegeneration affecting brain regions involved in CAN function [8]. Neuropathological studies have investigated the involvement of CAN regions in AD neurodegeneration, but to our knowledge no in vivo studies have used fMRI to study the relationship between CAN connectivity and early-stage Alzheimer’s pathophysiological change. To this end, the present study aims to evaluate the relationship between fMRI measures of resting-state CAN connectivity and plasma AD biomarkers. A relationship that if present could have significant clinical implications both as an imaging biomarker and as a potential therapeutic target for autonomic changes related to Alzheimer’s disease.

Descriptions of CAN in the literature are heterogeneous, and the brain regions associated with central autonomic function are diversifying [9] as investigations identify increasingly detailed regional task specificity [3, 10]. To account for this heterogeneity of CAN descriptions present in the literature, a CAN derived from a voxel-based meta-analysis of 43 studies was utilized for the present study [3], which will be referred to simply as the CAN. Studies of peripheral autonomic changes in AD and early stage cognitive dysfunction have specifically implicated the parasympathetic nervous system [5, 11, 12]. To disentangle sympathetic and parasympathetic contributions to the CAN, two function-specific CAN networks were also included from the same voxel-based meta-analysis. These include a parasympathetic CAN composed of brain regions associated with high frequency heart rate variability [3], and a sympathetic CAN composed of brain regions associated with electrodermal activity [3]. These networks will be referred to as the parasympathetic CAN and sympathetic CAN respectively. Sensorimotor network connectivity was included as a negative control to assess for potential global changes to functional connectivity associated with amyloid pathology. Also, default mode network (DMN) and hippocampal-posterior cingulate cortex (PCC) connectivity were controlled for to account for well-established functional connectivity changes associated with AD [13–18].

## Methods

### Participants

Participants were recruited from Orange County communities, and all procedures were conducted as part of the Vascular Senescence and Cognition (VaSC) Study at the University of California Irvine (UCI). Older adults aged 55 to 89 years who were living independently were included (Table 1). Exclusion criteria were history of clinical stroke, dementia, dysautonomia, major neurological or psychiatric disorder or medications impairing the central nervous system, current organ failure or other uncontrolled systemic illness, or contraindication for brain MRI. Study inclusions and exclusions were verified by a structured clinical health interview and review of current medications with the participant and, when available, a knowledgeable informant study partner. Participants underwent cognitive exams that included the Dementia Rating Scale (DRS) [19]. This study was approved by the UCI Institutional Review Board, and all participants gave informed consent. The anonymous data that support the findings of this study are available upon reasonable request from the corresponding author, DAN, through appropriate data sharing protocols.

## Measures

### *APOE* genotyping

Fasted blood samples were obtained by venipuncture and used to determine participant *APOE* genotype, as previously described [20]. Briefly, genomic DNA was extracted using the PureLink Genomic DNA Mini Kit (Thermo). The isolated DNA concentration was determined using a NanoDrop One (Thermo). DNA was then stored at − 80 °C for long-term storage. Isolated DNA was first diluted to a concentration of 10 mg/µL. PCR reactions were performed in a final volume of 25 µL containing 25 ng DNA, 0.5 µM of both forward and reverse primers (forward: ACGGCTGTCCAAGGAGCTG; reverse: CCCCGGCCTGGTACACTG), and 1× SYBR Green Master Mix (Qiagen) diluted in H2O. For the amplification, a T100 Thermal Cycler (BioRad) was used with the following settings: 95 °C for 10 min; 32 cycles of 94 °C for 20 s, 64 °C for 20 s, and 72 °C for 40 s; followed by 72 °C for 3 min. 15 µL of the DNA PCR product was digested with Hhal-fast enzyme at 37 °C for 15 min. The digested PRC product was added to a 3% agarose gel in 1× borax buffer for gel electrophoresis. The gel was run at 175 V for 25 min and visualized on ChemiDoc (BioRad) with a GelRed 10,000× gel dye. *APOE4* carrier status was defined as *APOE4* carriers (at least one copy of the ε4 allele) or *APOE4* non-carriers (no copies of the ε4 allele).

### MR imaging procedures

All participants underwent brain MRI scans conducted on a 3T Siemens Prisma scanner with 20-channel head coil. High-resolution 3D T1-weighted anatomical (Scan parameters: TR = 2300 ms; TE = 2.98 ms; TI = 900 ms; flip angle = 9 deg; FOV = 256 mm; resolution = 1.0 × 1.0 × 1.2 mm^3^; Scan time = 9 min) images were acquired, using 3-dimensional magnetization-prepared rapid gradient-echo (MPRAGE) sequences. Resting state fMRI scans comprised 140 contiguous echo-planar imaging (EPI) functional volumes (TR = 3,000 ms, TE = 30 ms, FA = 80°, 3.3 × 3.3 × 3.3 mm voxels, matrix = 64 × 64, FoV = 212 mm, 48 slices). T2-weighted scan parameters: TR = 10,000 ms; TE = 88.0 ms; flip angle = 120 deg; FOV = 210 mm; resolution = 0.8 × 0.8 × 3.5 mm^3^; Echo spacing = 9.8 ms; Echo trains per slice = 11; Scan time = 2 min).

### Selection of central autonomic network (CAN) regions of interest

The CAN chosen for this analysis was derived from a voxel-based meta-analysis of 43 fMRI autonomic task and resting state studies [3] that is generally associated with autonomic function (see ref. [3] Table 2) and two function-specific CAN networks, the parasympathetic CAN and the sympathetic CAN (see ref. [3] Table 3). The ROIs for all three networks were defined by 5 mm spheres around the MNI coordinates listed in Supplementary File 1; Table 1 along with connectivity analysis methodology using the CONN Functional Toolbox [21, 22]. DMN and hippocampal-PCC ROIs were defined using the default CONN toolbox parcellation [21].

### Plasma AD biomarkers

Blood plasma from fasted blood samples was separated by centrifugation and stored at -80 °C until AD biomarker assays. All plasma Aβ_40_ and Aβ_42_ concentrations were obtained using the digital immunoassay, Simoa Neurology 3-Plex A (N3PA) Advantage Kit (Quanterix). Plasma total tau was also obtained but not analyzed due to questions regarding its relationship with brain AD pathological changes [23]. Plasma levels of GFAP and NfL were determined using single molecule array, (Simoa®) Neurology 2-Plex B (N2PB) Kit (Quanterix), following the manufacturer’s protocol on the HD-X machine. Accepted ranges were as follows: NfL = 0 – ∼2000 pg/mL and GFAP = 0 – ∼40,000 pg/mL. All biomarker assays were conducted in the same lab at UCI.

### Data analysis

A total of 122 participants were characterized by demographics, resting state functional connectivity, and Aβ_42/40_ ratio data and were included for analysis. Plasma GFAP and NfL concentrations were obtained on a participant subset with available plasma (*n* = 94). Data was screened for outliers, and no participants met the criteria for removal (greater than ±3 SD from the mean). All adjusted models included age, sex, and *APOE4* carrier status as covariates unless otherwise noted.

First, linear regression analyses were conducted to determine the relationship between within-network functional connectivity and plasma AD biomarkers with and without demographic covariate adjustment. Hierarchical linear regression was also performed and Cohen’s F^2^ was assessed to quantify the additional effect of autonomic network connectivity on plasma AD biomarkers after accounting for demographic and genotypic covariates. Third, the Hayes Process Macro [24] model 1 was used to assess the moderating effects of *APOE4* carrier status with and without demographic covariate adjustment. For all identified significant relationships, two additional sensitivity analyses were performed using DMN functional connectivity and hippocampal – posterior cingulate cortex connectivity as covariates. This was done to ensure that the observed effects were specific to the autonomic networks under investigation rather than secondary to well established functional connectivity changes known to occur in AD.

The potential moderating effect of *APOE4* carrier status on the central autonomic networks-plasma AD biomarker relationships was also assessed. Lastly, to account for multiple comparisons, FDR correction [25] was performed.

## Results

122 older adults were included for analysis, participant characteristics and their demographics are displayed in Table 1.

**Table 1:** Participant characteristics, demographics, functional connectivity, and *APOE* genotype (*n* = 122)

Variable Name	Mean±SD
Age	69.50±7.34 (range 55–89)
Sex (n, female%)	84 (68.9)
*APOE2* carriers (n, 2/3%)	2 (1.6)
*APOE2* carriers (n, 2/4%)	2 (1.6)
*APOE3* homozygotes (n, 3/3%)	62 (50.8)
*APOE4* carriers (n, 3/4 or 4/4%)	50 (41.0)
*APOE4* homozygotes	4 (3.3)
Unknown or missing	2 (1.6)
Race/ethnicity	
Asian (n, %)	20 (16.4)
Native Hawaiian or pacific islander	0 (0)
American Indian or Alaska native	0 (0)
Black or African American	1 (0.8)
White	87 (71.3)
Other	2 (1.6)
Unknown or missing	12 (9.8)
Dementia Rating Scale	140±0.4
Aβ_42/40_ ratio	0.036±0.007
GFAP (pg/ml)	160.08±78.62
Neurofilament light chain (pg/ml)	19.28±8.05
CAN functional connectivity (RRC)	0.052±0.038
Parasympathetic CAN functional connectivity (RRC)	0.045±0.039
Sympathetic CAN functional connectivity (RRC)	0.036±0.033

CAN: Central autonomic network, RRC: fisher transformed ROI-to-ROI connectivity matrix, pg/ml: Picograms per milliliter, GFAP: Astrocytic intermediate filament glial fibrillary acidic protein

**Fig. 1:**
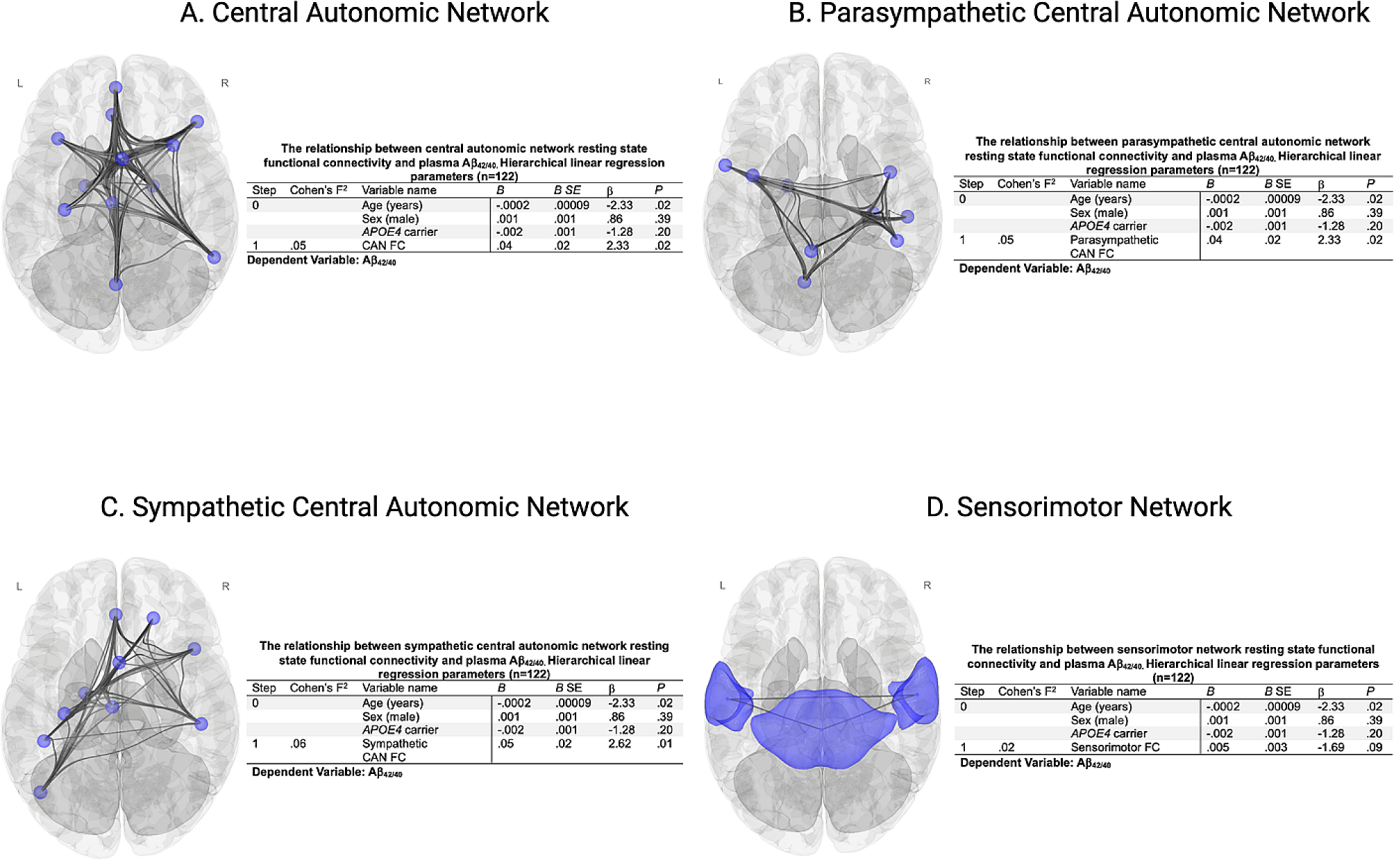
Hierarchical linear regression parameters quantifying the additional effect beyond demographic and *APOE4* carrier status of resting state within-network functional connectivity on plasma Aβ_42/40_ for the (**A**): Central Autonomic Network (CAN), (**B**): Parasympathetic CAN, (**C**): Sympathetic CAN, and (**D**): Sensorimotor network. (**B**): unstandardized regression coefficient, SE: standard error, β = standardized regression coefficient, FC: functional connectivity, *N* = 122

In univariate linear regression analyses, functional connectivity within the CAN (*B =* 0.05, *P =* .007), parasympathetic CAN (*B =* 0.04, *P =* .02), and sympathetic CAN (*B =* 0.06, *P =* .003) was positively associated with plasma Aβ_42/40_. Hierarchical linear regression analyses were performed to quantify the additional effect of within-network functional connectivity on plasma Aβ_42/40_ after adjustment for demographic factors and *APOE4* carrier status (Fig. 1A and C). The sensorimotor network was not associated with Aβ_42/40_ (Fig. 1D). No significant interaction between *APOE4* carrier status and CAN functional connectivity on Aβ_42/40_ was observed for any of the autonomic networks (CAN: *P* = .81, Parasympathetic CAN: *P* = .30, Sympathetic CAN: *P* = .68). *APOE4* carrier status was not associated with functional connectivity in any CAN network (CAN: *P* = .75, Parasympathetic CAN: *P* = .31, Sympathetic CAN: *P* = .43).

**Fig. 2:**
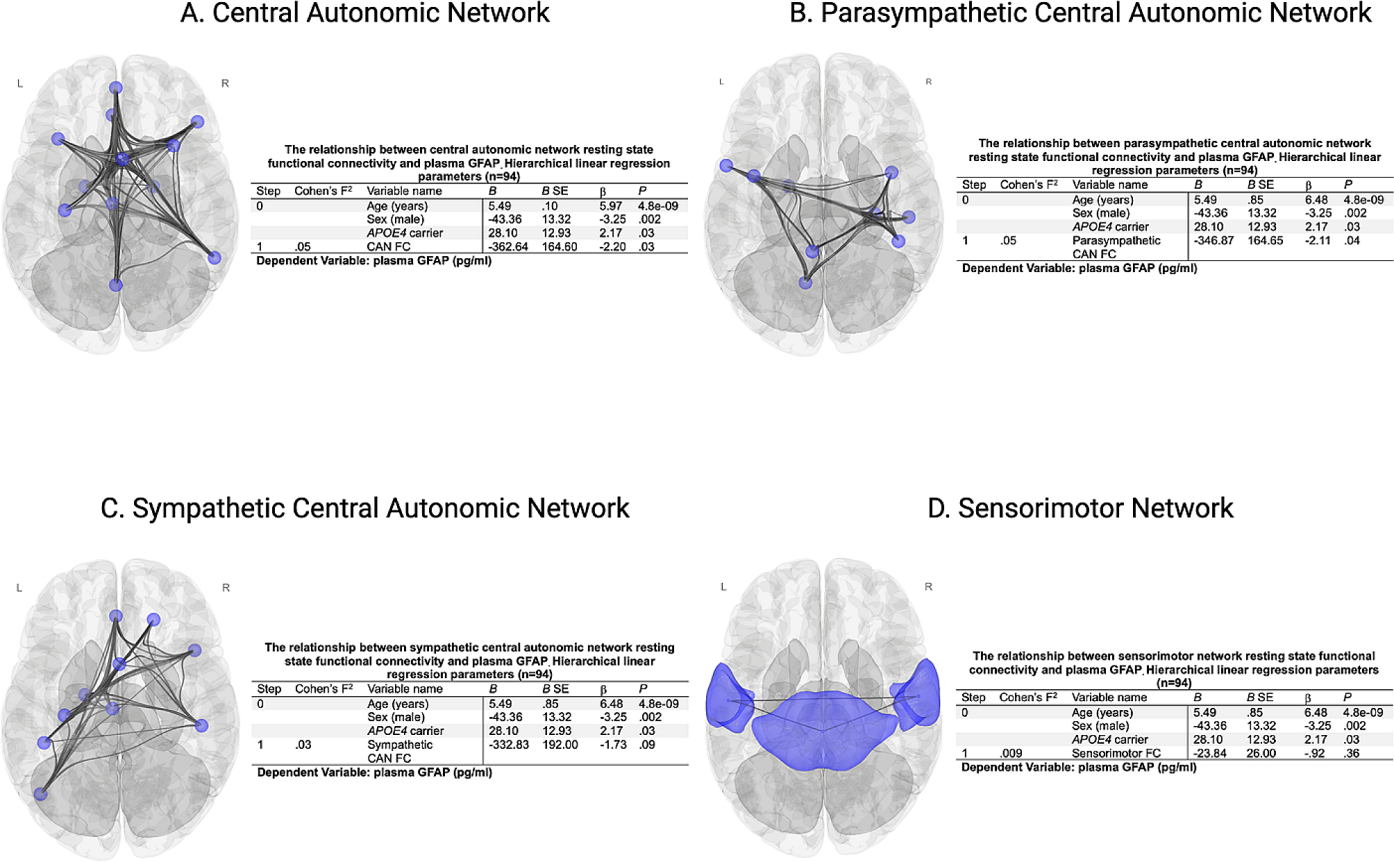
Hierarchical linear regression parameters quantifying the additional effect beyond demographic and *APOE4* carrier status of resting state within-network functional connectivity on fibrillary acidic protein (GFAP) in picograms per milliliter (pg/ml) for the (**A**): Central Autonomic Network (CAN), (**B**): Parasympathetic CAN, (**C**): Sympathetic CAN, and (**D**): Sensorimotor network. (**B**): unstandardized regression coefficient, SE: standard error, β = standardized regression coefficient, FC: functional connectivity, *N* = 94

**Fig. 3:**
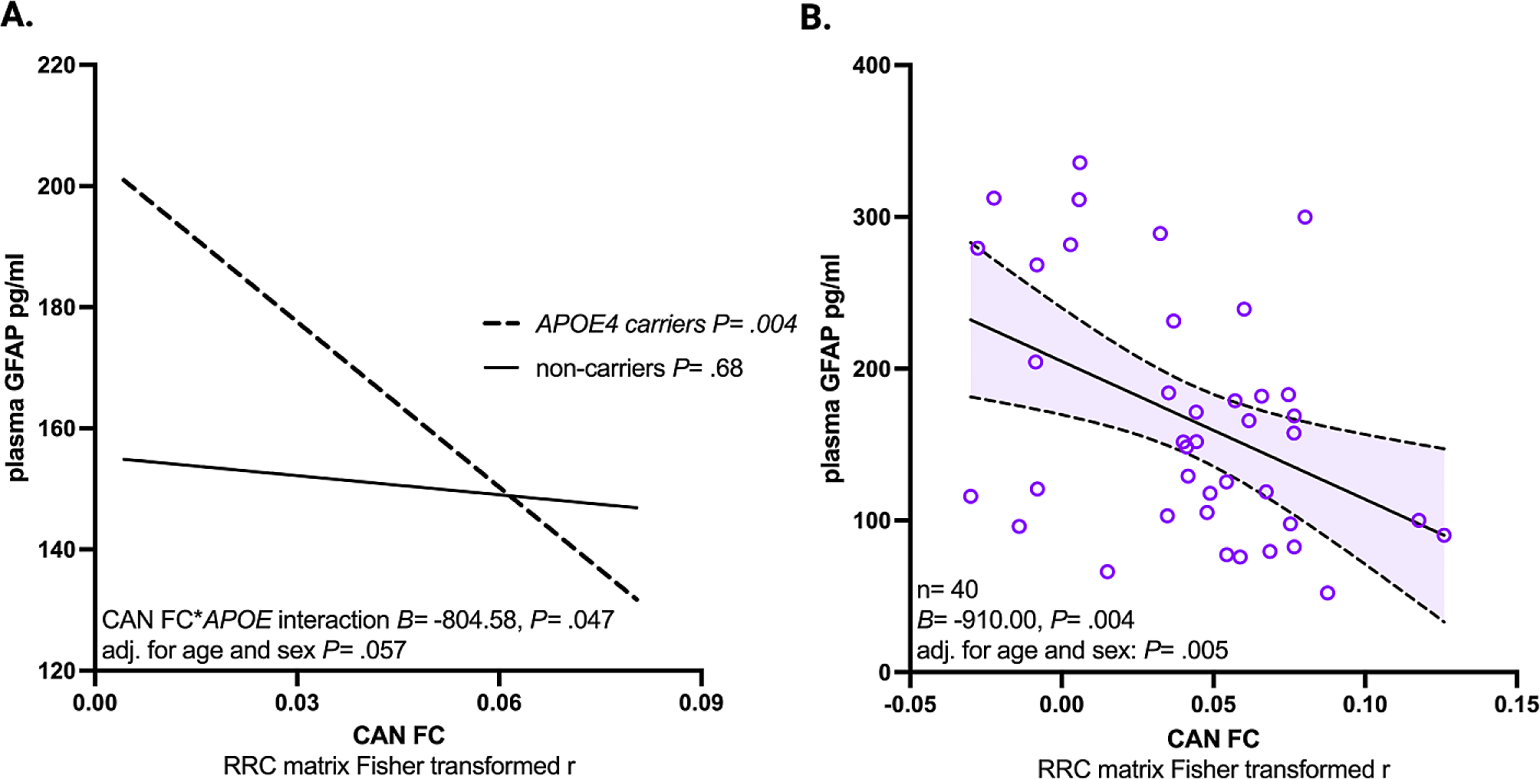
(**A**): The effect of parasympathetic central autonomic network (CAN) functional connectivity (FC) on glial fibrillary acidic protein in picograms per milliliter (GFAP pg/ml) conditional upon *APOE4* status is compared in 40 *APOE4* carriers and 54 non-carriers. (**B**): Scatterplot showing the relationship between resting state parasympathetic CAN FC and GFAP pg/ml in 40 *APOE4* carriers is shown and within group p-values are provided with and without demographic covariate adjustment

In univariate linear regression analyses, functional connectivity within the CAN (*B=* -576.84, *P =* .0037), parasympathetic CAN (*B=* -437.95, *P =* .031), and sympathetic CAN (*B=* -560.38, *P =* .02) was inversely associated with plasma GFAP. Hierarchical linear regression analyses were performed to quantify the additional effect of within-network functional connectivity on plasma GFAP after adjustment for demographic factors and *APOE4* carrier status (Fig. 2A-C). The sensorimotor network was not associated with plasma GFAP (Fig. 2D). A moderation analysis found no significant interaction between *APOE4* carrier status and functional connectivity on plasma GFAP for the general CAN (*P =* .81) or the sympathetic CAN (*P =* .68). However, parasympathetic CAN connectivity trended (*P* = .05) toward an interaction between *APOE4* carrier status, that when further investigated revealed parasympathetic CAN connectivity was inversely associated with GFAP concentration in *APOE4* carriers (*B*= -910.00, *P* = .004), but not in non-carriers (*B*= -105.43, *P* = .68) (Fig. 3A-B). The relationship between CAN parasympathetic functional connectivity and GFAP in *APOE4* carriers remained significant after adjustment for age and sex (*B=* -719.39, *P =* .005), age, sex, and DMN functional connectivity adjustment (*B=* -710.42, *P =* .007), and after age, sex, and hippocampus - posterior cingulate cortex functional connectivity adjustment (*B=* -747.12, *P =* .004).

**Fig. 4:**
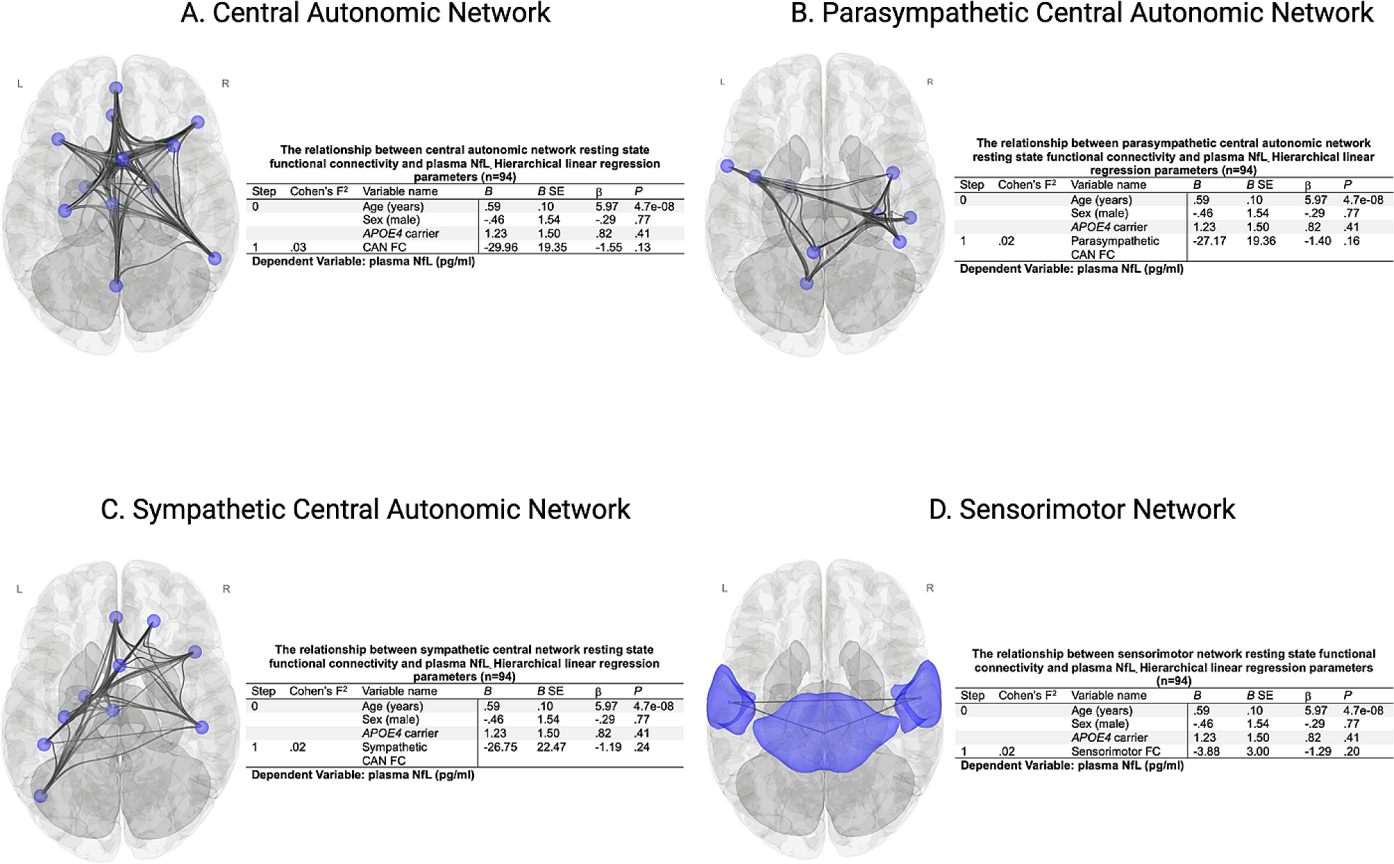
Hierarchical linear regression parameters quantifying the additional effect beyond demographic and *APOE4* carrier status of resting state within-network functional connectivity on plasma neurofilament light chain (NfL) in picograms/millimeter (pg/ml) for the (**A**): Central Autonomic Network (CAN), (**B**): Parasympathetic CAN, (**C**): Sympathetic CAN, and (**D**): Sensorimotor network. (**B**): unstandardized regression coefficient, SE: standard error, β = standardized regression coefficient, FC: functional connectivity, *N* = 94

Functional connectivity within the general CAN (*B=* -46.01, *P =* .04), but not the parasympathetic (*B=* -30.09, *P =* .18) or sympathetic (*B=* -46.95, *P =* .07) CANs, was negatively associated with plasma NfL, but this relationship was attenuated by adjustment for age, sex, and *APOE4* carrier status (Fig. 4A-C). Sensorimotor connectivity was not associated with NfL (Fig. 4D). No significant interaction between *APOE4* carrier status and functional connectivity on NfL was observed for any of the autonomic networks (CAN *P* = .68, Parasympathetic CAN *P* = .53, Sympathetic CAN *P* = .68).

The relationships between the three autonomic networks and the three plasma biomarkers (9 primary analyses) were included for FDR correction. Being negative controls, sensorimotor network analyses were not included. All seven significant findings survived FDR correction.

## Discussion

The present study is the first to investigate the association between central autonomic function and plasma AD biomarkers. Findings indicate decreased CAN connectivity was associated with plasma Aβ_42/40_ and GFAP in a sample of community-dwelling older adults with no history of stroke or dementia, suggesting that early changes in the brain’s autonomic control networks are associated with markers of early-stage Alzheimer’s pathophysiological change. Lower sympathetic and parasympathetic CAN connectivity were associated with plasma Aβ_42/40_, but only decreased parasympathetic CAN connectivity was related to plasma GFAP. This is consistent with peripheral autonomic studies suggesting parasympathetic changes in early-stage AD [5]. Plasma GFAP levels are indicative of astrocyte structural integrity loss [26, 27], central nervous system injury [26–28], impaired blood-brain barrier permeability [28], and early AD pathology [29–31]. In the present study, the relationship between GFAP and parasympathetic CAN connectivity was only observed in *APOE4* carriers, underscoring the relevance to AD risk. Previous research suggests that plasma GFAP is a sensitive marker of early cerebral amyloidosis [29], even in cognitively normal individuals with normal Aβ_42/40_ status [32]. This may explain the presence of an interaction effect on plasma GFAP but not Aβ_42/40_.

Plasma levels of NfL, a marker of neurodegeneration [33, 34], were not associated with CAN connectivity in either *APOE4* carriers or non-carriers, after statistically controlling for other factors. The lack of findings with NfL could be because our sample is relatively cognitively healthy and not expected to show substantial neurodegenerative changes at this stage. Future studies utilizing biomarkers of tau-mediated neurodegeneration may help clarify whether CAN connectivity is associated with early, AD-related neurodegeneration. Together the present study findings implicate central autonomic changes in the very early stages of AD pathophysiologic change, which may have major clinical implications.

Peripheral autonomic dysfunction is associated with AD, but it remains unclear whether these autonomic changes are symptomatic of AD, contributory to AD, or both [35]. The present study findings reveal for the first time that decreased connectivity within the CAN is associated with plasma AD biomarkers at an early stage. This finding implicates higher order autonomic regulation in early-stage Alzheimer’s pathophysiological changes, and could explain why prior studies observed peripheral autonomic dysfunction in AD. Consistent with prior peripheral autonomic studies implicating parasympathetic autonomic dysfunction in AD (e.g., decreased high-frequency HRV), we observed decreased connectivity in a parasympathetic network composed of high-frequency HRV-associated brain regions [3] associated with both plasma Aβ_42/40_ and GFAP levels. These findings have major implications since decreased HRV and the vagally-mediated arterial baroreflex are risk factors for early mortality [36, 37], and are major contributors to potentially harmful blood pressure variability changes [38, 39] that have previously been associated with higher risk for cerebrovascular disease [40], AD [41] and dementia [35]. Prior work has also specifically implicated increased blood pressure variability in AD risk among *APOE4* carriers [41], which is consistent with the present study findings associating higher plasma GFAP related to decreased parasympathetic CAN connectivity among *APOE4* carriers specifically. Future studies should further examine the role of higher order autonomic network changes in risk for cerebrovascular disease [42], AD and dementia.

The present study is cross-sectional and observational, precluding determination of causation or causal directionality. Very early neural network changes in older adults with cerebral amyloidosis may cause diminished CAN connectivity, ultimately leading to peripheral autonomic dysfunction. On the other hand, peripheral autonomic dysfunction may contribute to the AD process. Recent findings that several weeks of daily HRV biofeedback affects plasma Aβ levels suggest that peripheral autonomic activity influences Aβ production and/or clearance [43]. It is also possible that a third environmental or genetic factor is responsible for our observations. Future studies examining the effects of anti-amyloid treatments on CAN connectivity, or the effects of CAN connectivity modulation on cerebral amyloidosis (e.g., through transcranial magnetic stimulation), may help clarify the question of causality and directionality of our observations. These investigations should be conducted given the potential clinical relevance of autonomic dysfunction in early-stage AD.

Cerebral amyloid pathology on positron emission tomography has been previously associated with decreased functional connectivity early in AD progression [13], as well as in cognitively normal older adults [14]. This raises the possibility that the relationships between plasma AD biomarkers and network connectivity observed in the present study could be the result of previously well-established AD-related functional connectivity changes rather than being specifically related to CAN function. To address this question, we adjusted for connectivity within the DMN, a network traditionally associated with the earliest signs of AD-related functional connectivity decline [13, 15], which did not attenuate the observed relationships between plasma AD biomarkers and CAN connectivity. There are other early functional changes associated with prodromal AD, such as the functional isolation of the hippocampus [16–18]. Impaired connectivity between the hippocampus and posterior DMN nodes being the most pronounced, with a particularly prominent effect on hippocampal – PCC connectivity [17, 44–46]. Since both regions are CAN ROIs in the present analysis, and given the predilection for AD-related amyloid pathology in these regions [13, 47, 48], the possibility that the observed AD biomarker-CAN connectivity relationships are caused by general amyloid-related functional decline rather than CAN dysfunction is again raised. This is particularly true in the context of the observed *APOE4* modified relationship between plasma GFAP and parasympathetic CAN connectivity given the relationship between the *APOE4* genotype and functional connectivity, cerebral amyloid deposition, and AD risk. To control for this, hippocampal – PCC connectivity was also included as a covariate in the *APOE4* carrier status moderation analysis. Like the DMN, hippocampal functional isolation also did not attenuate the differential relationship between plasma GFAP and parasympathetic CAN connectivity in *APOE4* carriers. Lastly, to ensure that the observed changes were specific to the analyzed networks, the sensorimotor network was included as a negative control, and it displayed no significant relationships with any analyzed plasma AD biomarker. These additional control steps ensure that the observed relationship between parasympathetic CAN connectivity in *APOE4* carriers is specific to parasympathetic CAN function and not some other previously established marker of AD-related functional connectivity modification or a global change in functional connectivity associated with plasma AD biomarkers.

### Limitations

The examination of CAN connectivity based on a meta-analytic study [3] including both sympathetic and parasympathetic networks is a strength of the present study. In terms of limitations, it should be noted that CAN descriptions are increasingly diverse, context dependent, and task specific, likely reflecting spatial and task differentiation in CAN function [9]. Future studies should continue to investigate the relationship between the CAN and AD-related variables, including the analysis of function-specific CAN regions to further elucidate the complex and interconnected relationships between autonomic dysfunction and AD. Also, the broad age range and proximity to symptom onset of the present sample may hinder interpretation. Future studies could also benefit from including peripheral measures of autonomic function to assess the relationships between central and peripheral autonomic dysfunction with plasma AD biomarkers. Also, the present study’s cross-sectional design limits causal inference, and longitudinal studies are needed to understand the temporal relationships between AD neurodegeneration and autonomic dysfunction. Future studies investigating the relationship between peripheral autonomic markers and AD pathology should account for CAN connectivity to assess the potential for reverse causality or synergistic effects.

## Conclusion

Decreased CAN connectivity was associated with plasma AD biomarkers of cerebral amyloidosis (Aβ_42/40_) and brain injury (GFAP) suggesting that early changes in the brain’s autonomic control network are associated with early-stage Alzheimer’s pathophysiological change. The relationship between GFAP and parasympathetic CAN connectivity was only observed in *APOE4* carriers, underscoring the relevance to AD risk. Together the present study findings implicate central autonomic changes in the very early stages of AD pathophysiologic change, which may have major clinical implications and highlights the importance of additional studies of autonomic dysfunction in older adults at risk for cerebrovascular disease, AD, and dementia due to aging, genetics, and peripheral vascular risk factors.

### Electronic supplementary material

Below is the link to the electronic supplementary material.

Supplementary Material 1

## Data Availability

The anonymous data that support the findings of this study are available upon reasonable request from the corresponding author, DN, through appropriate data sharing protocols.
