# Supplementary material for: Central autonomic network dysfunction and plasma Alzheimer’s disease biomarkers in older adults
Source: Alzheimers Res Ther. 2024 Jun 8;16:124. doi: 10.1186/s13195-024-01486-9 (PMC11162037; doi:10.1186/s13195-024-01486-9)
Supplement: Supplementary file 1 — Supplementary Material 1 [file 13195_2024_1486_MOESM1_ESM.docx]

**Supplementary File 1: Resting state fMRI Central Autonomic Network Connectivity**

Functional and anatomical data were preprocessed using a flexible preprocessing pipeline in CONN (RRID:SCR_009550) release 22.a and SPM (RRID:SCR_007037) release 12.7771 (*2*), including realignment with correction of susceptibility distortion interactions, slice timing correction, outlier detection, direct segmentation and MNI-space normalization, and smoothing. Functional data were realigned using SPM realign & unwarp procedure (*3*), where all scans were co-registered to a reference image (first scan of the first session) using a least squares approach and a 6 parameter (rigid body) transformation, and resampled using b-spline interpolation to correct for motion and magnetic susceptibility interactions. Temporal misalignment between different slices of the functional data (acquired in interleaved Siemens order) was corrected following SPM slice-timing correction (STC) procedure (*4*), using sinc temporal interpolation to resample each slice BOLD timeseries to a common mid-acquisition time. Potential outlier scans were identified using ART as acquisitions with framewise displacement above 0.9 mm or global BOLD signal changes above 5 standard deviations (*5*), and a reference BOLD image was computed for each subject by averaging all scans excluding outliers. Functional and anatomical data were normalized into standard MNI space, segmented into grey matter, white matter, and CSF tissue classes, and resampled to 2 mm isotropic voxels following a direct normalization procedure (*6*) using SPM unified segmentation and normalization algorithm (*7*) with the default IXI-549 tissue probability map template. Last, functional data were smoothed using spatial convolution with a Gaussian kernel of 8 mm full width half maximum (FWHM).

In addition, functional data were denoised using a standard denoising pipeline including the regression of potential confounding effects characterized by white matter timeseries (5 CompCor noise components), CSF timeseries (5 CompCor noise components), motion parameters and their first order derivatives (12 factors) (*8*), outlier scans (below 75 factors) (*9*), session effects and their first order derivatives (2 factors), and linear trends (2 factors) within each functional run, followed by bandpass frequency filtering of the BOLD timeseries between 0.008 Hz and 0.09 Hz. CompCor (*10*) noise components within white matter and CSF were estimated by computing the average BOLD signal as well as the largest principal components orthogonal to the BOLD average, motion parameters, and outlier scans within each subject's eroded segmentation masks. From the number of noise terms included in this denoising strategy, the effective degrees of freedom of the BOLD signal after denoising were estimated to range from 19.2 to 163.3 (average 66.3) across all subjects.

ROI-to-ROI connectivity matrices (RRC) were estimated characterizing the patterns of functional connectivity within selected ROIs (**Table 1**). Functional connectivity strength was represented by Fisher-transformed bivariate correlation coefficients from a weighted general linear model (weighted-GLM (*11*)), defined separately for each pair of target areas, then averaged. To compensate for possible transient magnetization effects at the beginning of each run, individual scans were weighted by a step function convolved with an SPM canonical hemodynamic response function and rectified.

**Table 1:** Montreal Neurological Institute coordinates defining the central autonomic network (CAN) regions of interest centroids used in the present analysis (*1*).

| Central Autonomic Network ROI | x | y | z |
| --- | --- | --- | --- |
| Midcingulate Cortex | 2 | 10 | 40 |
| Thalamus (medial-dorsal nucleus, pulvinar), Superior colliculus/periaqueductal gray | -4 | -16 | 8 |
| Left amygdala/hypothalamus | -20 | -6 | -18 |
| Right anterior Insula | 32 | 18 | 6 |
| Right amygdala/hippocampal formation | 20 | -6 | -18 |
| Left anterior Insula | -36 | 22 | 0 |
| Ventromedial PFC, subgenual ACC | -4 | 36 | -24 |
| Pregenual ACC | -2 | 52 | -2 |
| Right angular gyrus, supramarginal gyrus | 56 | -48 | 22 |
| Ventral PCC, precuneous cortex, lingual gyrus | -2 | -64 | 10 |
| Left posterior Insula | -32 | -20 | 12 |
| Right frontoinsular cortex | 46 | 32 | -6 |
| Parasympathetic Central Autonomic Network ROI |  |  |  |
| Hippocampal formation | 30 | -22 | -16 |
| Amygdala, ventral tegmental area, hypothalamus | -20 | -6 | -18 |
| Anterior insula, caudate | -40 | 0 | 12 |
| Precuneus, dorsal posterior cingulate cortex | -6 | -44 | 34 |
| Primary motor cortex, temporal pole | -56 | 6 | 8 |
| Medial temporal gyrus, superior temporal gyrus | 50 | -24 | 2 |
| Supramarginal gyrus, angular gyrus | 44 | -38 | 14 |
| Cerebellum lobuli VI and vermis VI | -10 | -62 | -20 |
| Anterior insula | 40 | 2 | 12 |
| Sympathetic Central Autonomic Network ROI |  |  |  |
| Midcingulate cortex, paracingulate cortex, supplemental motor area | 0 | 10 | 40 |
| Supramarginal gyrus, superior parietal lobule, primary somatosensory cortex | 48 | -26 | 46 |
| Amygdala, subgenual ACC, nucleus accumbens, caudate, hippocampal formation | -20 | -8 | -12 |
| Ventromedial PFC, pregenual/subgenual ACC | -2 | 38 | -18 |
| Anterior insula, ventrolateral PFC | 44 | 18 | -6 |
| Thalamus (medial-dorsal nucleus), nucleus ruber, periaqueductal gray | -4 | -16 | 6 |
| Supramarginal gyrus, superior parietal lobe, primary somatosensory cortex | -44 | -36 | 42 |
| Secondary somatosensory cortex, posterior insula, putamen | -32 | -20 | 14 |
| Cerebellum lobulus crus I | -46 | -66 | -28 |
| Dorsolateral PFC | 20 | 36 | 34 |

PFC: prefrontal cortex, ACC: Anterior cingulate cortex, PCC: posterior cingulate cortex

1. F. Beissner, K. Meissner, K. J. Bär, V. Napadow, The autonomic brain: an activation likelihood estimation meta-analysis for central processing of autonomic function. *J Neurosci* **33**, 10503-10511 (2013).

2. S. Whitfield-Gabrieli, A. Nieto-Castanon, Conn: a functional connectivity toolbox for correlated and anticorrelated brain networks. *Brain Connect* **2**, 125-141 (2012).

3. J. L. R. Andersson, C. Hutton, J. Ashburner, R. Turner, K. Friston, Modeling Geometric Deformations in EPI Time Series. *NeuroImage* **13**, 903-919 (2001).

4. R. Sladky *et al.*, Slice-timing effects and their correction in functional MRI. *Neuroimage* **58**, 588-594 (2011).

5. J. D. Power *et al.*, Methods to detect, characterize, and remove motion artifact in resting state fMRI. *Neuroimage* **84**, 320-341 (2014).

6. V. D. Calhoun *et al.*, The impact of T1 versus EPI spatial normalization templates for fMRI data analyses. *Hum Brain Mapp* **38**, 5331-5342 (2017).

7. J. Ashburner, A fast diffeomorphic image registration algorithm. *NeuroImage* **38**, 95-113 (2007).

8. K. J. Friston, S. Williams, R. Howard, R. S. Frackowiak, R. Turner, Movement-related effects in fMRI time-series. *Magn Reson Med* **35**, 346-355 (1996).

9. M. N. Hallquist, K. Hwang, B. Luna, The nuisance of nuisance regression: spectral misspecification in a common approach to resting-state fMRI preprocessing reintroduces noise and obscures functional connectivity. *Neuroimage* **82**, 208-225 (2013).

10. X. J. Chai, A. N. Castañón, D. Ongür, S. Whitfield-Gabrieli, Anticorrelations in resting state networks without global signal regression. *Neuroimage* **59**, 1420-1428 (2012).

11. A. Nieto-Castanon, *Handbook of functional connectivity Magnetic Resonance Imaging methods in CONN*. (2020).
